# High Level of Plasma Estradiol as a New Predictor of Ischemic Arterial Disease in Older Postmenopausal Women: The Three-City Cohort Study

**DOI:** 10.1161/JAHA.112.001388

**Published:** 2012-06-22

**Authors:** Valérie Scarabin-Carré, Marianne Canonico, Sylvie Brailly-Tabard, Séverine Trabado, Pierre Ducimetière, Maurice Giroud, Joanne Ryan, Catherine Helmer, Geneviève Plu-Bureau, Anne Guiochon-Mantel, Pierre-Yves Scarabin

**Affiliations:** Centre for Research in Epidemiology and Population Health, Inserm Unit 1018, Hormones and Cardiovascular Disease, Villejuif, France (V.S., M.C., G.P.-B., P.-Y.S.); Université Paris-Sud, UMR-S 1018, Villejuif, France (V.S., M.C., P.D., P.-Y.S.); Service de Génétique moléculaire, Pharmacogénétique et Hormonologie, Hôpital Bicêtre, Assistance Publique–Hôpitaux de Paris, Le Kremlin-Bicêtre, France (S.B.-T., S.T., A.G.-M.); INSERM UMR-S693, Université Paris-Sud, Institut Fédératif de Recherche de Bicêtre, Bordeaux, France (S.B.-T., S.T., A.G.-M.); University Hospital and Faculty of Medicine of Dijon and EA 4184, University of Burgundy, Bordeaux, France (M.G.); INSERM Unit 1061, University Montpellier, Bordeaux, France (J.R.); INSERM Unit U897, Victor Segalen University, Bordeaux, France (C.H.); Université Paris Descartes, Paris, France (G.P.-B.)

**Keywords:** hormones, women, cardiovascular diseases, risk factors

## Abstract

**Background:**

Despite evidence that estrogens may be involved in atherothrombosis, the role of endogenous sex steroid hormones in ischemic arterial disease among postmenopausal women remains uncertain.

**Methods and Results:**

In the Three-City prospective cohort study of subjects (n=9294) >65 years of age, we investigated the association of total 17β-estradiol, bioavailable 17β-estradiol, and total testosterone with the 4-year incidence of ischemic arterial disease among postmenopausal women who did not use any hormone therapy. We designed a case–cohort study including a random sample of 537 subjects and 106 incident cases of first cardiovascular events. Weighted Cox proportional-hazards models with age as the time scale were used to estimate hazard ratios (HRs) and corresponding 95% confidence intervals (CIs) for ischemic arterial disease by a 1–standard deviation increase in sex steroid hormones. In univariate analysis, HR of ischemic arterial disease was positively and significantly associated with both total and bioavailable estradiol levels. These associations remained significant after adjustment for traditional cardiovascular risk factors, including body mass index, diabetes, hypercholesterolemia, hypertension, and smoking status (HR: 1.42, 95% CI: 1.12–1.79, *P*<0.01; and HR: 1.42, 95% CI: 1.12–1.78, *P*<0.01, respectively). Separate analysis for coronary heart disease yielded similar results (adjusted HR: 1.49, 95% CI: 1.10–2.02, *P*=0.01; and adjusted HR: 1.50, 95% CI: 1.11–2.04, *P*<0.01, respectively), and a borderline significant trend was observed for ischemic stroke (HR: 1.34, 95% CI: 0.95–1.89, *P*=0.08; and HR: 1.32, 95% CI: 0.94–1.84, *P*=0.11, respectively). By contrast, no significant association was found between total testosterone and ischemic arterial disease in both univariate and adjusted analyses.

**Conclusions:**

High plasma level of endogenous estradiol emerges as a new predictor of ischemic arterial disease in older postmenopausal women. **(*J Am Heart Assoc*. 2012;1:e001388 doi: 10.1161/JAHA.112.001388.)**

## Introduction

Incidence of coronary heart disease (CHD) is lower in women than men of the same age.^1,2^ The gap between the 2 sexes closes after menopause, when CHD rates rise sharply and cardiovascular disease becomes the leading cause of death among women. It therefore has been hypothesized that women's CHD advantage could be due to the protective effects of estrogens. Animal studies and observational studies have suggested that the use of postmenopausal hormone therapy (HT) could be beneficial with regard to the development of CHD.^3–5^ However, large prevention trials showed an increased risk of stroke in HT users and failed to confirm any estrogen-related cardioprotective effect.^6–8^ Nevertheless, a re-analysis of the Women's Health Initiative data recently has suggested that the timing of hormone initiation might influence the HT effect on CHD risk, with a decrease in CHD risk for women using HT close to menopause and a higher risk in older HT users.

Despite extensive biological research on the cardiovascular effects of estrogens,^9^ few studies have investigated whether endogenous sex steroid hormones (SSHs) could affect the risk of ischemic arterial disease among postmenopausal women. Previous data failed to provide evidence for an independent role of estradiol levels in determining CHD and stroke risk among postmenopausal women.^10–13^ Nevertheless, endogenous estradiol levels are positively related to cardiovascular risk factors such as obesity,^14^ dyslipidemia,^15–17 diabetes,18,19^ and C-reactive protein^20,21^ in postmenopausal women. In this context, we hypothesized that high levels of endogenous SSHs could be deleterious with regard to the risk of arterial disease among older postmenopausal women. Using the data from the Three-City (3C) cohort study, we therefore investigated the association of endogenous estradiol and testosterone with the risk of CHD and ischemic stroke among women >65 years of age.

## Methods

### Population Study

The 3C study is a large ongoing French prospective cohort study that aims to evaluate the risk of dementia attributable to vascular disorders. The study was approved by the Ethics Committee of the University Hospital of Kremlin-Bicêtre, and written informed consent was obtained from all participants. A detailed methodology of the study has been described previously.^22^ Briefly, 3649 men and 5645 women >65 years of age registered on electoral rolls and not institutionalized were recruited in 3 French cities (Bordeaux, Dijon, and Montpellier) between 1999 and 2001. Baseline data were collected by trained psychologists or nurses using standardized questionnaires during a face-to-face interview at home or at the study center. These data included information on sociodemographic characteristics, education, medical history, medication use, food consumption, and alcohol and tobacco use. Information on HT use was collected with a specific questionnaire. Women were classified as current HT users if they had used HT at any time during the 3 months before inclusion; otherwise they were classified as past users or never users. Systolic and diastolic blood pressure, weight, and height were assessed during a physical examination.

### Baseline Covariates

Smoking status was studied in 3 categories (never, past, and current). Body mass index (BMI) was calculated by dividing weight by height in meters squared. Hypertension status was defined as a high blood pressure measurement (systolic blood pressure ≥140 mm Hg and/or diastolic blood pressure ≥90 mm Hg), antihypertensive therapy at baseline, or both. Glycemia status was considered “diabetes” if the fasting glycemia value at inclusion was ≥1.26 g/L (7.00 mmol/L), the patient was receiving treatment for diabetes, or both. Glycemia status was “high glycemia” if the fasting glycemia value at inclusion was between 1.10 and 1.26 g/L (6.10 and 7.00 mmol/L) and was “normal glycemia” if the fasting glycemia value at inclusion was <1.10 g/L (6.10 mmol/L). Hypercholesterolemia was considered present if the cholesterol level was >2.40 g/L at baseline, the subject was treated for hypercholesterolemia, or both. Waist–hip ratio was calculated by dividing the waist circumference by the hip circumference.

### Follow-Up and Events Ascertainment

After baseline examination, subjects have been reexamined at home or at the study center every 2 years for the detection of cardiovascular events and dementia. For the present analysis, we used data collected over the 4-year follow-up.

Ischemic arterial disease consisted of either CHD or ischemic stroke during the follow-up. CHD was defined as a hospitalization for either stable or unstable angina pectoris, coronary dilatation, artery bypass, myocardial infarction, or definite CHD death. All CHD events were adjudicated by a medical committee. Nonfatal CHD events were validated by using hospital charts and practitioners’ reports. CHD deaths were validated by reviewing hospital records, medical data obtained from family physicians or specialists, and proxy interviews (coded I210 to I219, I251 to I259, I461, and R960 according to the *International Classification of Diseases*, 10th edition), as previously described.^23^ Stroke events were adjudicated within an independent group of experts and were defined as a rapid onset of a neurological deficit lasting >24 hours and confirmed by a lesion compatible with an acute stroke on computed tomography or magnetic resonance imaging of the brain. A review of brain imaging allowed further classification of strokes as ischemic or hemorrhagic events. The present analysis focuses on ischemic strokes. For subjects who presented both CHD and ischemic stroke during follow-up, we used the first cardiovascular event that occurred.

### Case–Cohort Study

Recently, a case–cohort study has been set up from the 3C study to investigate the association of blood biomarkers with cardiovascular risk and dementia. In brief, a case–cohort design consists of a random subsample of the original cohort together with all incident cases of this cohort. In practice, 1264 subjects were randomly selected from the initial cohort after stratification by study center, sex, age, and the presence of a baseline plasma sample. From the 759 selected women, we excluded women who were current HT users at inclusion (n=120). To investigate the risk of a first ischemic arterial disease event, we also excluded women with a personal history of CHD or stroke at inclusion (n=86). Finally, we excluded women without any follow-up data (n=16). Among these 537 remaining postmenopausal women, 15 incident cases of ischemic arterial disease were validated during the 4-year follow-up. With the same exclusion criteria applied, all incident cases outside the subcohort were added (n=91). The final population sample therefore consisted of 522 noncases and 106 incident cases of a first ischemic arterial disease event, including 67 CHD events and 39 ischemic strokes (Figure).

**Figure.**
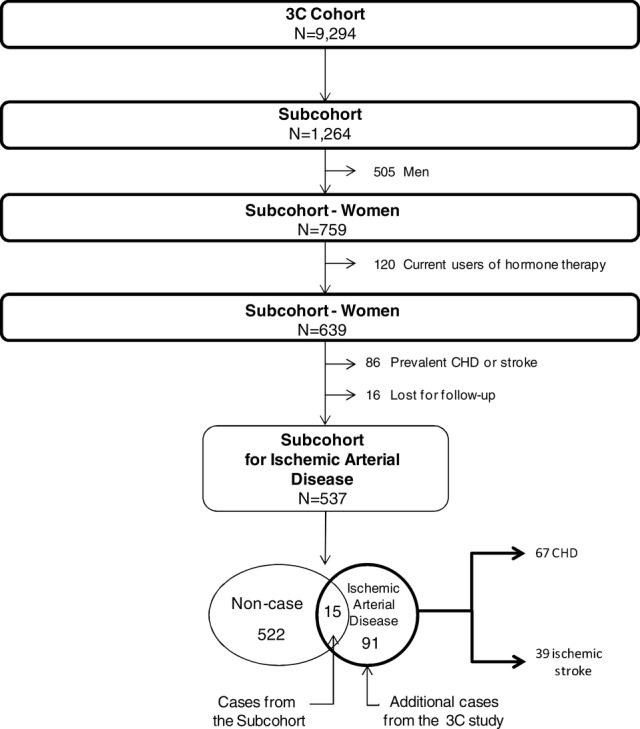
Flowchart representing the constitution of the case–cohort study for the investigation of the risk of ischemic arterial disease, CHD, and stroke in relation to sex hormone levels.

### Blood Collection and Hormone Measurements

At baseline, blood samples were collected for >90% of the full cohort. Plasma samples were available for all the subjects included in the case–cohort study. EDTA plasmas were obtained after 1 centrifugation at 3000*g* and were immediately stored at −80°C in 1-mL plastic tubes.

Plasma total estradiol was measured by a sensitive direct radioimmunoassay (RIA) with an Orion Diagnostica device (Spectria, Espoo, Finland). The minimum detectable concentration was 2 pg/mL (7.3 pmol/L), and a value of 0.6 pg/mL was arbitrarily assigned to all women with a nondetectable estradiol concentration. The intra-assay and interassay coefficients of variation were 17.6% and 18.1%, respectively, for a total estradiol concentration of 3.2 pg/mL (12 pmol/L) and were 2.8% and 5.8%, respectively, for a total estradiol concentration of 24 pg/mL. Bioavailable estradiol was assessed by differential precipitation of hormones bound to globulins with 50% ammonium sulfate after equilibration of the plasma sample with [^3^H]-estradiol and plasma total estradiol determination. Total and bioavailable estradiol were highly correlated to each other (*r*=0.98, *P*<0.01).

Plasma total testosterone was measured by a direct RIA as total estradiol on an Orion Diagnostica device (Spectria, Espoo, Finland). The minimum detectable concentration was 0.02 ng/mL (0.06 nmol/L), and the intra-assay and interassay coefficients of variation were 7.5% and 7.0%, respectively, for a total testosterone concentration of 0.46 and 0.35 ng/mL (1.6 and 1.2 nmol/L), respectively.

### Statistical Analyses

Baseline characteristics of subjects are displayed as frequencies for categorical variables and arithmetic means and standard deviations (SDs) for continuous variables that presented a normal distribution. Variables with a positively skewed distribution were log-transformed, and values were expressed as geometric means and interquartile ranges. Baseline characteristics of cases and noncases were compared by χ^2^ test and 2-tailed Student *t* test. The association of cardiovascular disease with total estradiol, bioavailable estradiol, and testosterone was assessed by using weighted Cox proportional-hazards models. Such models included a modification of the standard errors on the basis of robust variance estimates to take into account the case–cohort design, as previously described.^24,25^ In addition, because age is sharply associated to arterial disease, age was used as the time scale, as recommended.^26^ Each model presented met the proportional-hazards assumption. Hazard ratios (HRs) and 95% confidence intervals (95% CIs) were estimated for a 1-SD increase in the log-transformed SSH distribution. In addition, SSH distributions were divided into quartiles (Q1–Q4), and we estimated the risk of cardiovascular disease for each group compared with Q1 as the reference group. Tests for linear trend across the 4 categories of SSH levels were used to assess the significance of the variables in the models after having tested the linearity of the associations. To assess the linearity of the relation between SSH levels and ischemic arterial disease risk, we used tests based on the difference in the log-likelihood between 2 models of prediction (one with 3 dummy variables corresponding to the quartile of the parameter distribution, and the other including the qualitative ordinal variable in 4 categories). All tests were not significant, and thus we did not reject the hypothesis of linearity. Nonlinear (U-shaped) relations were also investigated with the use of quadratic terms in the Cox models. The risks of cardiovascular disease were assessed in an age-adjusted analysis and then were adjusted for study center and traditional cardiovascular risk factors (BMI, diabetes, hypercholesterolemia, hypertension, and smoking status). Finally, these multivariable analyses were further adjusted for waist–hip ratio. Data were missing for BMI and waist–hip ratio for 1% and 9% of subjects, respectively. Because adiposity is known to influence both the risk of ischemic arterial disease and estradiol levels, we used multiple imputations for missing data.^27^ The multiple-imputation prediction models included all variables in the conceptual framework. Five imputed data sets were created and analyzed together. The results were combined with Rubin's rules.^28^

Primary analyses focused on arterial ischemic disease, including both CHD and ischemic stroke. Then, subgroup analyses including only the first arterial event were performed separately for each vascular outcome. Using the same exclusion criteria among women free of arterial disease at baseline, we studied the risk of CHD among a subsample of 522 noncases and 67 incident cases. Similarly, the risk of ischemic stroke was estimated from a population consisting of 522 noncases and 39 incident events (Figure).

To determine whether certain subgroups of women were at particularly high or low risk for ischemic arterial disease, Cox proportional-hazards analyses were stratified according to the baseline levels of cardiovascular risk factors, and the consistency of hormone-related HRs was assessed by formal tests of interactions in the whole sample.

Statistical analyses were performed with the Statistical Analysis System software version 9.2 (SAS Institute Inc, Cary, NC).

## Results

Of the 106 women who experienced cardiovascular events, there were 25 myocardial infarctions, including 10 fatal events; 12 instances of angina pectoris; 25 coronary dilatations; 5 artery bypasses; and 39 ischemic strokes.

Characteristics of cases and noncases are presented in Table 1. Mean age at inclusion was higher among cases than noncase subjects (76.6 and 74.3 years, respectively). In addition, cases of ischemic arterial disease were more likely than noncases to have diabetes (20.7% and 6.7%, respectively) and hypertension (86.8% and 76.3%, respectively). With regard to SSH concentrations, the mean value of total estradiol was significantly higher among cases than noncases (6.05 and 5.19 pg/mL, respectively), and similar results were observed for bioavailable estradiol (4.04 and 3.48 pg/mL, respectively). By contrast, there was no significant difference in mean testosterone levels between cases and noncases (0.28 and 0.29 ng/mL, respectively).

**Table 1:** Baseline Characteristics and Plasma SSH Levels Among Cases of Ischemic Arterial Disease and Noncase Subjects in the 3C Case–Cohort Study

		Ischemic Arterial	
	Noncases	Disease Cases*	
Characteristic	(n=522)	(n=106)	*P*†
Age, y, mean±SD	74.3±5.3	76.6±5.7	<0.01

Study center, n (%)			0.38

Bordeaux	120 (23.0)	31 (29.3)	

Dijon	269 (51.5)	51 (48.1)	

Montpellier	133 (25.5)	24 (22.6)	

Education level, n (%)			0.55

Less than grade school	206 (39.5)	42 (39.6)	

Grade school or high school	154 (29.5)	36 (34.0)	

High school validated or university	162 (31.0)	28 (26.4)	

BMI‡, kg/m^2^, mean±SD	25.6±4.8	26.5±4.7	0.10

Glycemia§, n (%)			<0.01

Normal glycemia	466 (89.5)	80 (75.5)	

High glycemia	20 (3.8)	4 (3.8)	

Diabetes	35 (6.7)	22 (20.7)	

Hypertension, n (%)	398 (76.3)	92 (86.8)	0.02

Hypercholesterolemia, n (%)	330 (63.2)	70 (66.0)	0.58

Smoking, n (%)			0.83

Never	427 (81.8)	86 (81.1)	

Past	70 (13.4)	16 (15.1)	

Current	25 (4.8)	4 (3.8)	

Age at menopause‖, y, mean±SD	49.4±5.6	48.6±6.1	0.17

Type of menopause#, n (%)			0.74

Natural	421 (81.4)	84 (79.2)	

Bilateral oophorectomy	41 (7.9)	8 (7.6)	

Other	55 (10.7)	14 (13.2)	

SSHs, geometric mean (interquartile range)			

Total estradiol, pg/mL	5.19 (3.47–7.91)	6.05 (4.42–9.22)	<0.01

Bioavailable estradiol, pg/mL	3.48 (2.28–5.63)	4.04 (2.69–6.40)	<0.01

Total testosterone, ng/mL	0.29 (0.21–0.45)	0.28 (0.20–0.44)	0.49

* Including 67 CHD and 39 stroke events.

† *P* value obtained from Student *t* tests or χ^2^ tests except for SSHs, where Cox model was used.

‡ Two missed values

§ 1 missed value

‖ 6 missed values

# 5 missed values.

Table 2 shows the risk of ischemic arterial disease, CHD, and ischemic stroke in relation to total estradiol, bioavailable estradiol, and testosterone levels. In age-adjusted analysis, the risk of ischemic arterial disease was positively and significantly associated with total and bioavailable estradiol. HRs and 95% CIs for ischemic arterial disease per 1-SD increase in total and bioavailable estradiol distribution were 1.39 (1.13–1.72) and 1.38 (1.12–1.70), respectively, and subjects with the highest levels of estradiol (Q4) had an increased risk of ischemic arterial disease as compared to subjects with the lowest levels of estradiol (HR: 1.99, 95% CI: 1.06–3.73, *P* for linear trend=0.02 for total estradiol; and HR: 2.22, 95% CI: 1.18–4.20, *P* for linear trend=0.02 for bioavailable estradiol). Adjustment for study center and traditional cardiovascular risk factors, including BMI, diabetes, hypercholesterolemia, hypertension, and smoking status, slightly attenuated these associations. HRs and 95% CIs for a 1-SD increase were 1.42 (1.12–1.79) and 1.42 (1.12–1.78) for total and bioavailable estradiol, respectively, and HRs and 95% CIs of Q4 versus Q1 were 1.99 (0.96–4.10) and 2.19 (1.05–4.56) for total and bioavailable estradiol, respectively. Further adjustment for waist–hip ratio made no substantial changes to the results: 1.37 (1.07–1.74) and 1.36 (1.07–1.73) for total and bioavailable estradiol, respectively. Separate analysis for CHD (n=67 events) yielded similar results (adjusted HR: 1.49, 95% CI: 1.10–2.02, *P*=0.01; and adjusted HR: 1.50, 95% CI: 1.11–2.04, *P*<0.01, respectively), and a borderline significant trend was observed for ischemic stroke (n=39 events) (HR: 1.34, 95% CI: 0.95–1.89, *P*=0.08; and HR: 1.32, 95% CI: 0.94–1.84, *P*=0.11, respectively). Stratified analyses showed similar HRs for ischemic arterial disease by cardiovascular risk factors, including BMI, diabetes, hypercholesterolemia, and hypertension. For example, no risk modification related to obesity or diabetes was found. The HRs (95% CIs) of ischemic arterial disease for a 1-SD increase of total estradiol were 1.26 (1.02–1.55) and 1.28 (0.79–2.08) in the nonobese and obese (BMI >30 kg/m^2^) women, respectively, and 1.21 (0.99–1.49) and 1.37 (0.78–2.39) in the nondiabetic and diabetic women, respectively.

**Table 2:** HRs of Ischemic Arterial Disease, CHD, and Stroke Events by SSH Level in Postmenopausal Women of the 3C Case–Cohort Study

	Ischemic Arterial Disease*							CHD							Ischemic Stroke						
			
		Age Adjusted			Adjusted‡				Age Adjusted			Adjusted‡				Age Adjusted			Adjusted‡		
															
SSH Level	No. of Events	HR	95% CI	*P*†	HR	95% CI	*P*†	No. of Events	HR	95% CI	*P*†	HR	95% CI	*P*†	No. of Events	HR	95% CI	*P*†	HR	95% CI	*P*†
Total estradiol, pg/mL																					

For 1 SD log	106	1.39	(1.13–1.72)	<0.01	1.42	(1.12–1.79)	<0.01	67	1.43	(1.11–1.86)	<0.01	1.49	(1.10–2.02)	0.01	39	1.35	(0.95–1.92)	0.08	1.34	(0.95–1.89)	0.08

Q1 <3.51	19	1	[reference]	0.02	1	[reference]	<0.05	12	1	[reference]	0.07	1	[reference]	0.12	7	1	[reference]	0.10	1	[reference]	0.13

Q2 [3.51–5.27]	20	1.33	(0.66–2.66)		1.46	(0.70–3.09)		14	1.46	(0.62–3.42)		1.55	(0.61–3.93)		6	1.09	(0.35–3.35)		1.30	(0.40–4.26)	

Q3 [5.27–7.83]	31	1.71	(0.91–3.23)		1.84	(0.90–3.76)		19	1.71	(0.77–3.78)		1.72	(0.70–4.25)		12	1.75	(0.67–4.58)		2.17	(0.73–6.46)	

Q4 ≥7.83	36	1.99	(1.06–3.73)		1.99	(0.96–4.10)		22	1.98	(0.90–4.35)		2.08	(0.81–5.32)		14	2.00	(0.77–5.15)		1.95	(0.70–5.42)	

Bioavailable estradiol, pg/mL																					

For 1 SD log	106	1.38	(1.12–1.70)	<0.01	1.42	(1.12–1.78)	<0.01	67	1.45	(1.11–1.88)	<0.01	1.50	(1.11–2.04)	<0.01	39	1.30	(0.93–1.82)	0.13	1.32	(0.94–1.84)	0.11

Q1 <2.32	18	1	[reference]	0.02	1	[reference]	0.05	12	1	[reference]	0.05	1	[reference]	0.10	6	1	[reference]	0.18	1	[reference]	0.20

Q2 [2.32–3.56]	25	1.83	(0.92–3.63)		2.03	(0.96–4.26)		15	1.66	(0.70–3.94)		1.76	(0.68–4.54)		10	2.56	(0.91–7.19)		2.93	(0.98–8.76)	

Q3 [3.56–5.49]	26	1.73	(0.89–3.35)		1.90	(0.90–3.98)		16	1.58	(0.70–3.58)		1.63	(0.64–4.15)		10	1.84	(0.63–5.34)		2.38	(0.77–7.38)	

Q4 ≥5.49	37	2.22	(1.18–4.20)		2.19	(1.05–4.56)		24	2.20	(1.01–4.80)		2.26	(0.89–5.69)		13	2.26	(0.83–6.16)		2.22	(0.76–6.52)	

Total testosterone, ng/mL																					

For 1 SD log	106	0.93	(0.76–1.14)	0.49	0.91	(0.72–1.15)	0.42	67	0.91	(0.72–1.15)	0.44	0.89	(0.67–1.18)	0.42	39	0.95	(0.68–1.33)	0.76	0.93	(0.65–1.40)	0.69

Q1 <0.23	34	1	[reference]	0.76	1	[reference]	0.33	22	1	[reference]	0.60	1	[reference]	0.42	12	1	[reference]	0.77	1	[reference]	0.61

Q2 [0.23–0.33]	21	0.71	(0.39–1.30)		0.67	(0.36–1.26)		12	0.61	(0.28–1.31)		0.56	(0.25–1.27)		9	0.98	(0.38–2.55)		0.98	(0.35–2.70)	

Q3 [0.33–0.45]	25	1.03	(0.56–1.87)		0.95	(0.49–1.85)		17	0.98	(0.49–1.98)		0.95	(0.42–2.14)		8	0.81	(0.30–2.22)		0.85	(0.31–2.29)	

Q4 ≥0.45	26	0.83	(0.47–1.47)		0.70	(0.37–1.32)		16	0.73	(0.36–1.48)		0.61	(0.26–1.40)		10	0.92	(0.38–2.24)		0.81	(0.31–2.08)	

* Including 67 CHD and 39 stroke events.

† *P* value for continuous variable and *P* for trend for variable in quartiles.

‡ Model adjusted for study center, BMI, diabetes, hypertension, hypercholesterolemia, and smoking status.

Finally, we conducted sensitivity analyses with exclusion of past HT users (102 noncases and 18 cases), women who started HT during the follow-up (2 noncases and 1 case), or events that occurred during the first year of follow-up (21 cases). Overall, no substantial change in the association of SSH levels with ischemic arterial disease was observed (data not shown).

With regard to testosterone, both age-adjusted and fully adjusted analyses showed no significant association with the risk of ischemic arterial disease (for 1-SD increase in testosterone distribution: HR: 0.93; 95% CI: 0.76–1.14; and HR: 0.91, 95% CI: 0.72–1.15, respectively). There was no significant U-shaped relationship between testosterone and the risk for ischemic arterial disease. Total testosterone was not associated with the risk of CHD and ischemic stroke separately.

## Discussion

To our knowledge, this is the first study to show a positive association between plasma estradiol levels and the risk of both CHD and ischemic stroke among postmenopausal women >65 years of age. These associations were independent of traditional cardiovascular risk factors such as diabetes and BMI. By contrast, plasma levels of testosterone were not significantly related to the risk of arterial ischemic disease.

Few studies have evaluated the association between endogenous estrogens and risk of ischemic arterial disease. The Rancho Bernardo Study of 651 postmenopausal women with a mean age of 66 years who were not using HT failed to show any association of total and bioavailable estradiol levels with the risk of death from ischemic heart disease.^10^ Nevertheless, a recent study found that elevated endogenous estradiol levels could predict risk of all-cause death in older postmenopausal women.^29^ However, cardiovascular death was not investigated in this study. In a nested case–control design among postmenopausal women not using HT, crude and adjusted analyses based on 115 incident cases of combined ischemic arterial disease, similar to our primary outcome, showed no association between estradiol levels and cardiovascular risk.^11,13^ More recently, another cohort study of 99 CHD events reported an increased risk of CHD among women with high estradiol concentrations. Nevertheless, this association disappeared after adjustment for BMI and other cardiovascular risk factors.^13^ In these 2 latter studies, postmenopausal women were younger than those included in our cohort, and this difference in age, as well as a lack of statistical power, may partly explain the diverging results in the association of SSH levels with CHD risk. Finally, our findings are consistent with the recent results from a prospective study that reported a positive association between the free estradiol index and the risk of atherothrombotic stroke (196 incident cases) among postmenopausal women >65 years of age.^12^ However, potential mediators included dyslipidemia and insulin resistance, whereas our data showed an independent association between elevated estradiol levels and ischemic arterial disease.

The existence of an independent association between high levels of endogenous estradiol and the risk of ischemic arterial disease among older women does not necessarily imply that this relationship is causal. Nevertheless, estradiol may affect several mechanisms that may be involved in atherothrombosis. Adipose aromatization of testosterone represents the main source of estradiol production in women after cessation of ovarian activity. Therefore, obese women were more likely than lean ones to present high levels of estradiol.^14,30,31^ Both adipose tissue and high levels of endogenous estradiol have been associated with a low-grade inflammation state that can be a mechanism for mediating the association of estradiol with ischemic arterial disease.^20,32,33^ In our study, adjustment for BMI and waist–hip ratio did not substantially modify the association of estradiol with cardiovascular disease. Nevertheless, BMI and waist–hip ratio may be an indirect assessment of fat mass, and therefore, we cannot exclude this mechanism. Further prospective data including baseline measurement of fat mass are required. Insulin resistance and diabetes also could explain our results because both have been consistently related to high levels of endogenous estradiol among postmenopausal women without HT.^18,19,34^ However, in our study, adjustment for diabetes slightly modified the increased cardiovascular risk associated with high endogenous estradiol levels. Changes in the lipid profile could be another mechanism for mediating the association of estradiol with the risk of arterial ischemic disease. However, data on the effects of endogenous estradiol on lipids remain somewhat conflicting. Although early investigations suggested a positive association of estradiol levels with high-density lipoprotein cholesterol,^35^ recent findings have indicated a deleterious lipid profile among postmenopausal women with the highest levels of estradiol. Indeed, it has been shown that estradiol and estrone were positively associated with triglycerides,^16^ low-density lipoprotein cholesterol,^17^ and total cholesterol^15^ and were negatively associated with high-density lipoprotein cholesterol.^16^ Finally, procoagulant effects of estrogens may represent a central mechanism in the increased risk of cardiovascular disease in postmenopausal women with high estradiol levels. It recently has been shown that thrombin generation time, a hypercoagulability marker, could have an important role in the etiology of ischemic arterial disease, especially among women in the elderly.^36,37^ Thus, we can hypothesize that the association of estradiol with ischemic arterial disease is partly mediated by deleterious changes in hemostasis. However, data relating endogenous estradiol levels and hemostatic variables in postmenopausal women are required. Overall, better understanding of the mediators of estradiol effects is still needed, and further mechanisms remain to be investigated, especially with regard to the effect of endogenous estradiol on atherogenesis and endothelial function in older postmenopausal women.

Data on the role of testosterone in cardiovascular disease are scarce and remain conflicting. One study has found that low levels of total testosterone were an independent risk factor for CHD among postmenopausal women.^38^ In addition, other data showed that low levels of testosterone could predict both all-cause and cardiovascular death among older women.^39^ By contrast, the Women's Health Study reported that a high free androgen index was related to an increased risk of ischemic arterial disease in postmenopausal women without HT, but this association was not independent of traditional cardiovascular risk factors.^11^ More recently, the Cardiovascular Health Study reported an association of high testosterone levels with the risk of CHD among older postmenopausal women.^40^ In addition, the same investigation showed that testosterone was positively associated with some cardiovascular risk factors, such as insulin resistance and metabolic syndrome.^40^ This latest result was concordant with an earlier study that found a positive and significant association of free testosterone with the degree of coronary artery disease among postmenopausal women.^41^ In our study, neither age-adjusted nor fully adjusted analyses displayed an association between testosterone and the risk of ischemic arterial disease in older women. We cannot exclude the possibility that the inconsistency in previous findings and absence of association in our study could be due to heterogeneity in vascular disease outcomes, population characteristics (eg, age, health status), and hormone measurements between studies.

Our study has several strengths. First, the 3C study is a prospective and multicenter study with a high participation rate during the 4 years of follow-up. In addition, baseline data, including detailed information on HT use, were collected by standardized questionnaires during a face-to-face interview. Moreover, hormone measurements were conducted without knowledge of the case/noncase status via a RIA method, giving only 8% of values under the minimum detectable concentration.^42^ Finally, incident cases of CHD and stroke were carefully validated by 2 independent committees of experts using medical documentation.

The main limitation of our study is the small number of incident cases, which may yield a lack of statistical power, especially for subgroup analyses and for testing potential interactions of SSH levels with cardiovascular risk factors on ischemic arterial disease. In addition, this study included older women with a high prevalence of cardiovascular risk factors, especially hypertension, and therefore our results cannot be generalized to healthy younger postmenopausal women. With regard to the SSH assays, especially at low levels of estradiol in postmenopausal women, conventional RIAs with preceding purification steps would provide more reliable and accurate measurements of plasma estradiol as compared with direct RIA.^43^ However, measurement error related to direct RIA would bias our analysis toward the null hypothesis, resulting in a potential underestimation of the true associations.

In conclusion, a high level of endogenous estradiol emerges as a new significant predictor of the risk of ischemic arterial disease among postmenopausal women older than 65 years. However, further investigations are needed to confirm these results and to assess the role of endogenous SSHs in cardiovascular disease, especially in younger postmenopausal women without cardiovascular risk factors.
